# 1100. Cellular Immunophenotypic Predictors of Influenza Vaccine Immunogenicity in Pediatric Hematopoietic Cell Transplant Recipients

**DOI:** 10.1093/ofid/ofad500.073

**Published:** 2023-11-27

**Authors:** Justin Z Amarin, Joshua Simmons, Haya Hayek, Daniel Dulek, Andrew J Spieker, James Chappell, Natasha B Halasa, Spyros A Kalams

**Affiliations:** Vanderbilt University Medical Center, Nashville, Tennessee; Vanderbilt University Medical Center, Nashville, Tennessee; Vanderbilt University Medical Center, Nashville, Tennessee; Vanderbilt University Medical Center, Nashville, Tennessee; Vanderbilt University Medical Center, Nashville, Tennessee; Vanderbilt University Medical Center, Nashville, Tennessee; Vanderbilt University Medical Center, Nashville, Tennessee; Vanderbilt University Medical Center, Nashville, Tennessee

## Abstract

**Background:**

Pediatric hematopoietic cell transplant recipients (PHCTRs) have poor serologic responses to influenza vaccination. While absolute numbers of circulating B and T cells are important predictors of response, our goal was to identify B and T cell subpopulations associated with vaccine immunogenicity.

**Methods:**

We conducted a secondary analysis of PHCTRs enrolled in a multicenter randomized controlled influenza vaccine trial spanning three influenza seasons (2016–2019). Peripheral blood mononuclear cells were collected before vaccination and 28–42 days after the second dose, and they were analyzed using mass cytometry (CyTOF). We fit linear regression models to estimate relationships between strain-specific (A/H3N2, A/H1N1, and B/Victoria) pre- to post-vaccination (28–42 days) serum hemagglutination-inhibition antibody titers vs. log-transformed cell counts, including vaccine dose as a covariate. We used the Benjamini–Yekutieli method to adjust for multiplicity. We conducted a subsequent sensitivity analysis, including time from transplant as an additional covariate, for cell populations identified as predictive of response to all three antigens.

**Results:**

We included 156 PHCTRs. The median age at first vaccination was 11.8 years (IQR, 6.8–14.3 years), and the median time from transplantation was 7.9 months (IQR, 4.3–13.6 months). High-dose trivalent or standard-dose quadrivalent inactivated influenza vaccine was administered to 79 (50.6%) and 77 (49.4%) PHCTRs, respectively. Mass cytometry revealed 33 distinct cell subpopulations, seven of which were significant predictors of geometric mean titer ratio for all three antigens after two vaccine doses, irrespective of dose (Table 1). We found evidence that subpopulations 1 and 18 (related B cell subpopulations differing by BTLA and CCR6 expression), 8 (CD8+ naïve T cells), and 19 (CD4+ T follicular helper cells) predicted titers irrespective of dose and time from transplantation.

Table 1

**Figure:**
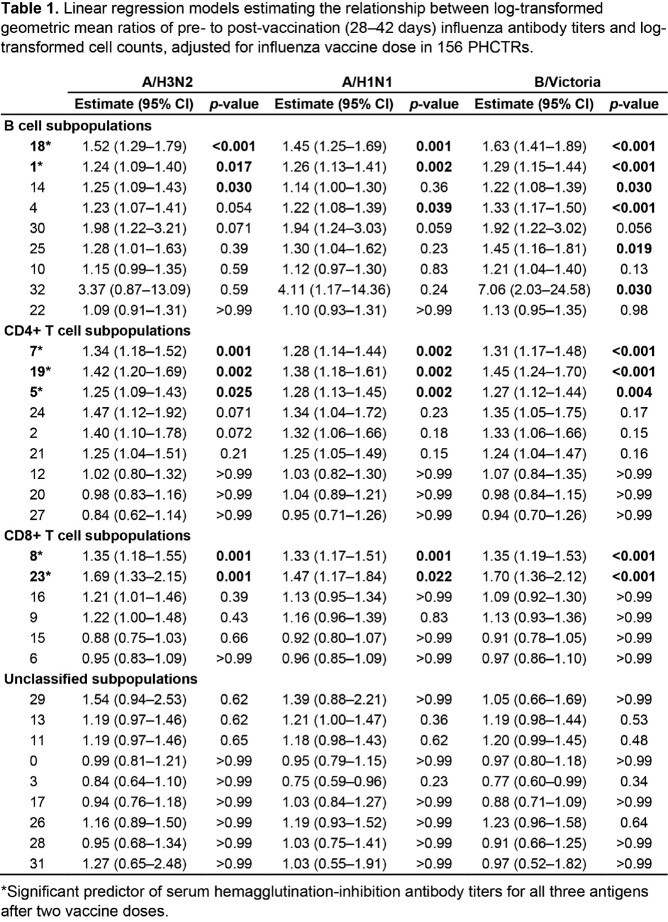

Figure 1

**Figure:**
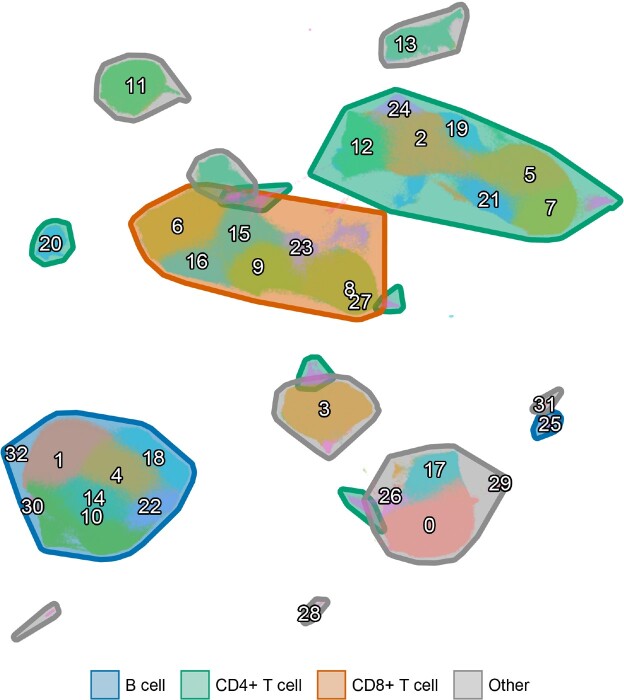

UMAP from a 32-parameter CyTOF antibody panel generated from 156 PHCTRs at the time of vaccination. Major islands of CD4+ T cells, CD8+ T cells, and B cells are demarcated.

Figure 2

**Figure:**
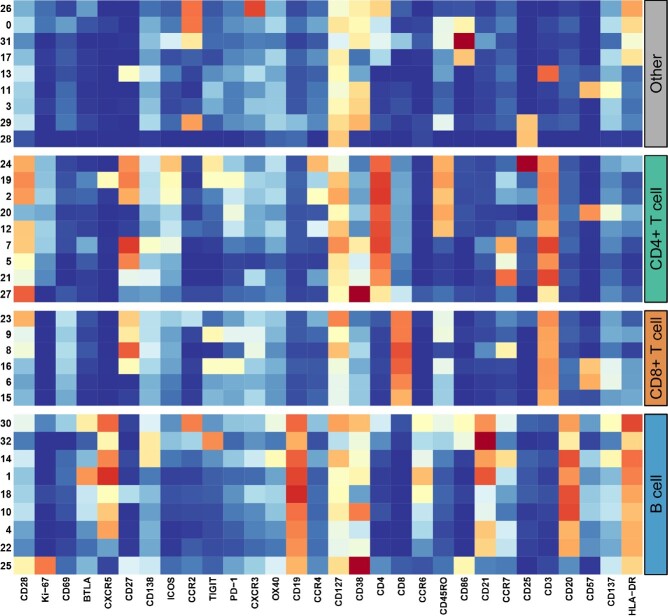

Heat map of cell subpopulations identified by CyTOF. The frequency of several cell subpopulations predicted subsequent serological responses to multiple vaccine immunogens, including 1 and 18 (B cells), 5 and 7 (naïve CD4+ T cells), 19 (memory cTfh cells), and 23 (CD8+ memory T cells).

**Conclusion:**

We identified distinct B and T cell subpopulations predictive of influenza vaccine immunogenicity in PHCTRs. Our findings may improve the identification of PHCTRs at high-risk for influenza due to impaired influenza vaccine immunogenicity and aid in the development of effective vaccine strategies in this population.

**Disclosures:**

**Daniel Dulek, MD**, Eurofins Viracor: Research supplies **Natasha B. Halasa, MD, MPH**, Merck: Grant/Research Support|Quidell: Grant/Research Support|Quidell: donation of kits|Sanofi: Grant/Research Support|Sanofi: vaccine support

